# Defining the Role of Adjuvant Radiotherapy for Biliary Tract Cancers: A Site-Specific Propensity-Matched Analysis

**DOI:** 10.3390/cancers17030494

**Published:** 2025-02-02

**Authors:** Yongwoo David Seo, Belkacem Acidi, Andrew Newton, Antony Haddad, Yi-Ju Chiang, Rainna Coelho, Timothy E. Newhook, Ching-Wei D. Tzeng, Yun Shin Chun, Ethan B. Ludmir, Eugene J. Koay, Milind Javle, Jean Nicolas Vauthey, Hop S. Tran Cao

**Affiliations:** 1 Department of Surgical Oncology, The University of Texas MD Anderson Cancer Center, Houston, TX 77030, USA; dseo@mcw.edu (Y.D.S.); acidi.belkacem@gmail.com (B.A.);; 2 Department of Surgical Oncology, Ochsner Health, New Orleans, LA 70112, USA; 3 Department of Surgery, HCA Houston Healthcare, University of Houston, Houston, TX 77204, USA; 4 Department of Radiation Oncology, The University of Texas MD Anderson Cancer Center, Houston, TX 77030, USAekoay@mdanderson.org (E.J.K.); 5 Department of Gastrointestinal Medical Oncology, The University of Texas MD Anderson Cancer Center, Houston, TX 77030, USA

**Keywords:** biliary tract cancer, adjuvant therapy, chemoradiotherapy, liver cancer

## Abstract

**Background**: Biliary tract cancers (BTCs) have distinct tumor biology but share a poor prognosis, with a 5-year-survival-rate of 5–19%. Surgical resection is the only potential cure, but recurrences are common. The role of adjuvant radiotherapy (XRT) remains unclear. **Methods**: Using the National Cancer Database (2006–2018), we analyzed resected non-metastatic BTCs. Patients who survived beyond 90 days post-surgery were included, while those with R2 resections or neoadjuvant therapy were excluded. Propensity matching was performed based on predictors of adjuvant radiation, age, and sex. Survival outcomes were compared between no adjuvant therapy, chemotherapy alone, and XRT ± chemotherapy. **Results**: Among 21,275 patients, including 5308 intrahepatic cholangiocarcinoma (IHC), 2689 perihilar cholangiocarcinoma (PHC), 3092 distal cholangiocarcinoma (DCC), and 10,186 gallbladder cancer (GBC) cases, adjuvant XRT did not improve survival for IHC. For PHC and DCC, XRT improved survival over no adjuvant therapy (PHC: 31.2 vs. 26.3 months, *p* = 0.004; DCC: 33.7 vs. 27.0 months, *p* = 0.015) but not over chemotherapy alone. For GBC, XRT significantly improved survival compared to both no adjuvant therapy and chemotherapy (30.2 vs. 26.6 and 24.6 months; *p* = 0.05 and *p* = 0.001). **Conclusions**: XRT provides a survival benefit for GBC, especially in node-positive and R1-resected patients. For PHC and DCC, XRT improves outcomes compared to no therapy, but its benefit over chemotherapy is uncertain. No benefit was observed for IHC.

## Simple Summary

Biliary tract cancers represent a group of rare malignancies with a generally poor prognosis, even after potentially curative surgery. Additional treatments such as chemotherapy and radiotherapy are often used to improve survival, but their effectiveness varies depending on the cancer type. In this study, we analyzed data to determine the impact of radiotherapy for different biliary cancer subtypes. We found that adjuvant radiotherapy significantly improves survival for gallbladder cancer, particularly in patients with lymph node involvement or incomplete tumor removal (R1). For bile duct cancers, radiotherapy is beneficial compared to no treatment but offers limited advantages over chemotherapy alone. For intrahepatic cholangiocarcinoma, no survival benefit was observed with radiotherapy. These findings emphasize the need for tailored treatment strategies based on the specific type of biliary tract cancer.

## 1. Introduction

Biliary tract cancers represent a group of rare malignancies with some commonalities but also distinct tumor biology that includes intrahepatic cholangiocarcinoma, perihilar cholangiocarcinoma, distal cholangiocarcinoma, and gallbladder cancer. Surgical resection is the only potentially curative treatment for all biliary tract cancers; however, even with complete resection, both distant and locoregional recurrences are common. Overall, prognosis is poor, with a 5–19% five-year survival rate [1,2,3,4,5].

The best adjuvant treatment for resected biliary tract cancers is unclear. The only phase III clinical trial to demonstrate a survival benefit with adjuvant therapy for resected biliary tract cancers was the BILCAP trial [6]. In this randomized controlled trial of 447 patients, overall survival was superior with 6 months of adjuvant capecitabine compared to observation alone; thus, recommendations are for 6 months of adjuvant capecitabine following surgical resection. A potential benefit of adjuvant chemoradiation was suggested by the phase II trial SWOG S0809 [7]. In this single-arm trial, 79 patients with extrahepatic cholangiocarcinoma and gallbladder carcinoma treated with curative intent resection received adjuvant gemcitabine and capecitabine followed by concurrent capecitabine and radiation. The 2-year overall survival rate of 65% compared favorably to historical outcomes. However, S0809 has yet to been confirmed with a follow-up phase III clinical trial. In addition, given the relative rarity of biliary tract malignancies, most retrospective and prospective studies measure composite outcomes for some combination of the disease sites despite their biologic differences.

Furthermore, if adjuvant therapy is beneficial for resected biliary tract cancers, it is unknown which subgroups of patients might benefit the most. This lack of clarity is reflected in the NCCN guidelines for resected biliary tract cancers [8], which list adjuvant chemoradiation as an option for patients with resected intrahepatic cholangiocarcinoma and positive margins or regional lymph nodes and for all patients with resected extrahepatic cholangiocarcinoma or gallbladder cancer.

Given the relative paucity of data and the tendency to group multiple disease sites together in previous studies, our goal was to determine the disease site-specific benefit of adjuvant radiation for resected intrahepatic cholangiocarcinoma, perihilar cholangiocarcinoma, distal cholangiocarcinoma, and gallbladder cancer.

## 2. Materials and Methods

### 2.1. Data

This was a retrospective study using the National Cancer Database (NCDB) 2006–2018 Liver Participant Use File and Gallbladder and Extrahepatic Bile Duct Participant Use File. The NCDB is a large, prospective, hospital-based cancer registry that collects and reports data on approximately 70% of all patients with newly diagnosed cancers at over 1500 Commission on Cancer-accredited centers in the United States. It is a joint project of the American College of Surgeons’ Commission on Cancer and the American Cancer Society [9]. Patients with extrahepatic cholangiocarcinoma were separated into perihilar and distal tumors using site-specific code 25 in the NCDB. This study was approved by the Institutional Review Board at The University of Texas MD Anderson Cancer Center (2022–0512).

### 2.2. Patient Selection

Patients aged ≥18 years with resected non-metastatic invasive intrahepatic cholangiocarcinoma, perihilar cholangiocarcinoma, distal cholangiocarcinoma, and gallbladder cancer were included. Patients who died within 90 days of surgery, those with R2 resections, and those who received neoadjuvant chemotherapy and/or neoadjuvant radiation were excluded. Patients with stage 1 gallbladder cancer (T1aN0M0) were also excluded as current guidelines recommend simple cholecystectomy alone as the definitive treatment.

### 2.3. Propensity Matching and Survival Analysis

Categorical variables were reported as frequency (percentage) and continuous variables were reported as median (interquartile range (IQR)). Baseline characteristics and outcomes were compared using the Pearson’s x2 or Fisher’s exact test for categorical variables and the Wilcoxon rank-sum test for continuous variables. Logistic regression was used to identify predictors of adjuvant radiation for each disease site. Patients were then propensity matched 1:1 on predictors of adjuvant radiation (*p* ≤ 0.05), age, and sex. Overall survival with no adjuvant treatment, adjuvant chemotherapy alone, or adjuvant radiation ± chemotherapy was compared using Kaplan–Meier estimates. The association of treatment strategy with overall survival stratified by margin and lymph node status was also compared using Cox regression.

Patients were propensity matched 1:1 based on the likelihood of receiving adjuvant radiation, with a logistic regression model informing the selection of variables. This model included significant predictors of adjuvant radiation (*p* ≤ 0.05), along with age and sex, to compute the propensity scores. Matching was then conducted utilizing a nearest-neighbor algorithm with a caliper width of 0.2 standard deviations of the logit of the propensity score, ensuring comparable groups and reducing selection bias. This matching was applied to two distinct sets of pairwise comparisons for each biliary disease subtypes, focusing on overall survival: first comparing surgery alone to surgery with adjuvant (chemo)radiation (PSM Set 1) and then comparing adjuvant chemotherapy to adjuvant (chemo)radiation (PSM Set 2). The four disease subtypes stratified were intrahepatic cholangiocarcinoma (IHC), perihilar cholangiocarcinoma (PHC), distal cholangiocarcinoma (DCC), and gallbladder adenocarcinoma (GBC). Further stratified analyses were conducted based on margin and nodal status to elucidate subgroup benefits.

## 3. Results

**Figure 1:**
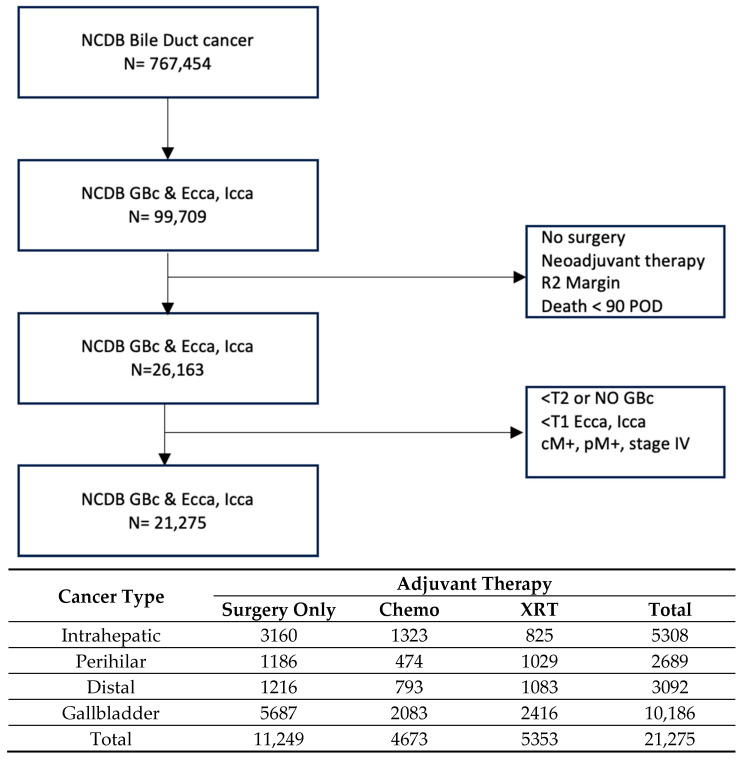
Flow diagram and population distribution. NCDB: National Cancer Database; GBC: gallbladder cancer; ECCA: extrahepatic cholangiocarcinoma; ICCA: intrahepatic cholangiocarcinoma; POD: post-operative days. Chemo: chemotherapy; XRT: chemo(radiation) therapy.

In total, there were 21,275 patients in the entire cohort; the patient breakdown by cancer type was as follows: 5308 IHC, 2689 PHC, 3092 DCC, and 10,186 GBC (Figure 1). Patients within these groups were then categorized by adjuvant therapy type received: no adjuvant; chemotherapy only; and (chemo)radiation, which will be labeled XRT for the rest of this manuscript.

In order to perform the propensity score matching analysis of adjuvant therapy, two univariate comparator groups were selected within each cancer type: no adjuvant therapy vs. adjuvant XRT (PSM Set 1) and adjuvant chemotherapy vs. adjuvant XRT (PSM Set 2). The patient numbers utilized within each cancer type were 1446 and 1382 for IHC, 1386 and 890 for PHC, 1452 and 1444 for DCC, and 4110 and 3536 for GBC for PSM Sets 1 and 2, respectively (Table 1, Supplementary Tables S1–S4).

Propensity-matched survival analyses are shown in Table 2. For the overall cohort of patients with IHC, no difference in survival was noted for either PSM Set 1 or PSM Set 2. Amongst the PHC and DCC cohorts, adjuvant XRT was associated with improved survival compared with no adjuvant therapy (for PHC: median OS 31.2 months with XRT vs. 26.3 months with no adjuvant therapy, *p* = 0.004; for DCC: median OS 33.7 months with XRT vs. 27.0 months with no adjuvant therapy, *p* = 0.015), but not compared to adjuvant chemotherapy. Amongst patients with GBC, there was a statistically significant benefit of XRT compared to both no adjuvant therapy (median OS 30.2 months with XRT vs. 26.6 months with no adjuvant therapy vs. XRT, *p* = 0.05) and adjuvant chemotherapy (median OS 30.2 months with adjuvant XRT vs. 24.6 months with adjuvant chemotherapy, *p* = 0.001) (Table 2).

## 4. Subset Analyses

**Figure 2:**
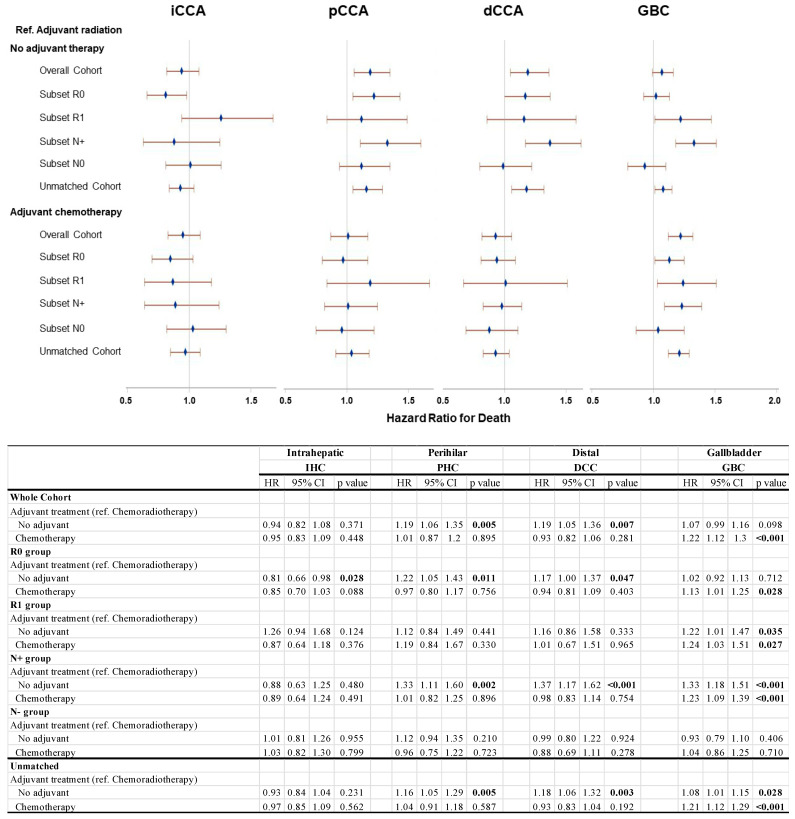
Sensitivity analysis of propensity-matched cohorts using Cox proportional hazards model.

For each disease site, subset analyses were performed focusing on margin status (R0/R1) and nodal status (N+/N0). In the GBC patient cohorts, adjuvant XRT was associated with a significantly improved survival advantage in nearly all evaluated subsets when contrasted with both no adjuvant therapy and adjuvant chemotherapy. Specifically, relative to adjuvant XRT, surgery alone showed an increased hazard ratio for death at 1.07 (95% CI 0.99–1.16) for the entire matched cohort. This difference was most pronounced following R1 resection (HR 1.22 (95% CI 1.01–1.47)) and for patients with positive lymph nodes (HR1.33 (95% CI 1.18–1.51)). Comparatively, relative to adjuvant XRT, adjuvant chemotherapy was associated with an HR for death of 1.22 (95% CI 1.12–1.30) for the whole matched cohort. The HR was 1.13 (95% CI 1.01–1.25) with R0 resection and 1.24 (95% CI 1.03–1.51) for R1 resection; it was 1.23 (95% CI 1.09–1.39) for N+ disease (Figure 2).

Amongst patients with PHC and DCC, there was a similar pattern of significantly improved outcomes with adjuvant XRT when compared to no adjuvant therapy (PSM Set 1) but not against adjuvant chemotherapy (PSM Set 2); these effects were seen in the whole matched cohort and the R0 and the N+ sub-groups, but not in the R1 and N0 groups. In both PHC and DCC, the largest effect size was seen in the N+ subset; in PHC, the HR for death was 1.33 (95% CI 1.11–1.60), while in DCC, the HR was 1.37 (95% CI 1.17–1.62) with no adjuvant therapy vs. adjuvant XRT (Figure 2).

Across all patient subsets with IHC, there was no apparent survival benefit to adjuvant XRT when compared to surgery alone or adjuvant chemotherapy, even in the presence of R1 resection status (Figure 2).

## 5. Discussion

As it has become increasingly evident, BTC are composed of distinctly separate diseases with disparate biologic profiles, behavior, and treatment responses. In this study, through propensity score matched models using a large dataset, we sought to define the role of XRT across individual BTC, finding a differential impact of this adjunctive modality depending on the disease site. We find that XRT is associated with a significant survival benefit in GBC. For PHC and DCC, XRT appears to improve outcomes when compared to no adjuvant therapy, although its benefit over chemotherapy alone remains uncertain. Conversely, adjuvant XRT was not associated with a survival advantage for IHC.

For GBC, we find in this propensity score matched analysis that XRT can improve survival across the board, but especially for patients with N+ disease or margin-positive resection. Importantly, XRT appeared to confer a benefit even when compared to chemotherapy alone. These findings are in line with results from the GECCOR-GB trial, which support the effectiveness of adjuvant radiotherapy for resected GBC. This multicenter, randomized phase II study reported a 1-year disease-free survival rate of 77.8% with capecitabine concurrent with radiotherapy that met the minimum pre-specified DFS rate for a positive trial [10]. Additionally, a recent meta-analysis by Choi et al. reported that XRT, while not universally beneficial for all GBC patients, can be particularly effective for those with specific clinical profiles, such as node-positive or margin-positive disease, the same cohorts in our present study who appeared to derive the greatest benefit from adjuvant XRT [11].

The use of XRT in BTC can take various forms, and we should also mention internal radiotherapy, such as Yttrium-90 radioembolization (Y90), which is currently under investigation. Y90 has shown promise in downstaging unresectable intrahepatic cholangiocarcinoma to allow for curative surgery, although its role in the adjuvant setting remains less well defined [12].

While our data point to a clear benefit of XRT in GBC, the advantages of XRT in PHC and DCC are less definitive, especially when compared to chemotherapy alone. Achieving negative surgical margins is paramount for PHC, which suggests that the optimal benefit of adjuvant therapies, including XRT, might be contingent on such surgical outcomes [13,14]. Given the complexity of these cancer subtypes, a nuanced approach to management is required. Future investigations should aim to delineate more clearly the contexts in which XRT could be considered advantageous over chemotherapy, potentially involving novel therapeutic combinations or stratification based on molecular and radiologic biomarkers.

Additionally, XRT has been studied as part of protocols designed to bridge patients with BTC to liver transplantation. For perihilar cholangiocarcinoma, protocols such as those developed at the Mayo Clinic, which combine neoadjuvant XRT with liver transplantation, have reported promising survival outcomes, highlighting the potential of radiotherapy in carefully selected patients [15].

It is important to note that the inclusion of patients with RX margins (~17%) in our propensity-matched cohort represents a limitation, as RX status may confound survival outcomes due to its significant prognostic impact. Future studies should account for margin heterogeneity (R0, R1, and RX) to better define the role of adjuvant therapies. With regards to IHC, our study finds no significant survival advantage with the use of XRT when compared to surgery alone or adjuvant chemotherapy, even in cases with R1 resection status (Figure 2). This observation aligns with the broader consensus that XRT’s role in IHC is limited. Although the application of selective internal radiotherapy (SIRT) alongside chemotherapy has shown potential in downstaging unresectable IHC, thus enabling surgical intervention in certain cases, its effectiveness as an adjuvant therapy has yet to be confirmed [16].

Nearly a decade ago, SWOG 0809 reported a 2-year survival rate of 65% and a median overall survival of 35 months for patients with extrahepatic cholangiocarcinoma and GBC treated with adjuvant chemoradiation, outcomes that surpassed historical results that at the time were achieved in the absence of any known effective adjuvant therapy [7]. Recently, Dominguez et al. validated these findings by conducting a NCDB study in which the authors matched the inclusion criteria of their study cohort to those of SWOG 0809 [17]. They reported a nearly identical median overall survival of 36.9 months in patients receiving chemoradiation. Our present study largely corroborates the findings from both publications but adds further insight into our understanding of the role of XRT for biliary tract cancers in some critical ways. First, whereas PHC and DCC were grouped together under the category of extrahepatic cholangiocarcinoma, which could obscure differences in treatment responses between these cancer types, we made sure to consider and analyze them separately. Additionally, neither SWOG 0809 nor the recent contribution by Dominguez evaluated XRT for IHC, a disease site for which our study found no significant survival advantage with adjuvant XRT.

Finally, it bears mentioning that a number of studies have previously examined the role of neoadjuvant XRT in BTC. For instance, Kobayashi et al. reported that the three-year recurrence-free survival (RFS) rates in patients treated with neoadjuvant chemoradiation therapy using full-dose gemcitabine combined with 50–60 Gy of radiation prior to resection of biliary tract cancers were 78%, compared to 58% in those treated without neoadjuvant therapy (*p* = 0.0263). The study included patients with PHC, GBC, and DCC and supported the potential of neoadjuvantly delivered XRT to improve surgical outcomes and long-term survival [18]. Similarly, TOSBIC02 was a phase I study that investigated neoadjuvant S-1 plus cisplatin with concurrent radiation in patients with resectable stage II-IVa extrahepatic cholangiocarcinoma. It reported promising results, with 33% (3 patients with PHC and 1 with DCC) of patients achieving a partial response and another 33% (4 patients with DCC) maintaining stable disease. Of the 12 patients enrolled, 9 underwent radical surgery, achieving an R0 resection rate of 58% [19]. However, the study was terminated early due to high morbidity and unexpected mortality rates, highlighting the need for careful patient selection and management of treatment-related toxicities. Due to the heterogeneity of patients who might have required neoadjuvant radiation across the disease sites, we opted to focus our study on adjuvant XRT only.

Our study has several inherent limitations, typical of analyses conducted using large databases. Firstly, we did not delve into the specifics of radiation therapy, such as the modality used or the total radiation dose/intensity, which could potentially influence treatment outcomes. Additionally, as the landscape of BTC treatment evolves with the integration of immune checkpoint inhibitors, as evidenced by TOPAZ-1 [20] and KEYNOTE-966 [21], the durability of our findings remains uncertain. Furthermore, the National Cancer Database lacks detailed data on adjuvant therapy, including specific chemotherapy and radiation regimens. While efforts were made to mitigate this limitation by employing propensity-matched cohorts and strict selection criteria, there’s a possibility that patients received varying treatment regimens that could bias our results. Despite these limitations, our study provides valuable insights into the subtype-specific benefits of adjuvant radiotherapy for BTC, paving the way for future research.

## 6. Conclusions

In our propensity-matched analyses of patients undergoing potentially curative resection for biliary tract cancers, we demonstrate that the addition of chemoradiotherapy in the adjuvant setting seems most beneficial in patients with gallbladder cancer, especially those who have node positive disease or R1 resection margins. Conversely, and consistent with prior studies, there appears to be no role for radiation after curative-intent surgical resection of IHC. For PHC and DCC, some form of adjuvant therapy is indicated—whether XRT adds much to chemotherapy alone remains debatable. These findings lend a rationale for more rigorous, high quality, prospective trials of the impact of XRT within specific biliary tract cancers to determine who stands to benefit the most from adjuvant chemoradiotherapy.

## Figures and Tables

**Table 1:** Propensity-matched groups, all biliary tract cancers. Patients were then propensity matched 1:1 on predictors of adjuvant radiation (*p* ≤ 0.05), age, and sex. Chemo: chemotherapy; XRT: chemo(radiation) therapy.

All Biliary Tumors	Matched Group 1 (n = 8394)				Matched Group 2 (n = 7252)			
	Surgery Only (n = 4197)		XRT (n = 4197)		Chemo (n = 3626)		XRT (n = 3626)	
	n	Col %	n	Col %	n	Col %	n	Col %
**SEX**								
**Male**	1824	43.5%	1866	44.5%	1562	43.1%	1578	43.5%
**Female**	2373	56.5%	2331	55.5%	2064	56.9%	2048	56.5%
**Age**								
**≤65**	1813	43.2%	1811	43.1%	1743	48.1%	1725	47.6%
**>65**	2384	56.8%	2386	56.9%	1883	51.9%	1901	52.4%
**Race**								
**White**	3067	73.1%	3009	71.7%	2616	72.1%	2562	70.7%
**Black**	421	10.0%	480	11.4%	389	10.7%	432	11.9%
**Hispanic**	382	9.1%	392	9.8%	324	8.9%	364	10.0%
**Asian/Pacific Islander**	275	6.6%	240	5.7%	224	6.2%	214	5.9%
**Others**	52	1.2%	55	1.3%	52	1.4%	48	1.3%
**Margin**								
**R0**	2782	66.3%	2788	66.4%	2434	67.1%	2430	67.0%
**R1**	684	16.3%	691	16.5%	548	15.1%	563	15.5%
**RX**	731	17.4%	718	17.1%	644	17.8%	633	17.5%
**Pathologic T Stage**								
**T1**	281	6.7%	321	7.6%	212	5.8%	233	6.4%
**T2**	1594	38.0%	1548	36.9%	1225	33.8%	1243	34.3%
**T3**	1436	34.2%	1405	33.5%	1379	38.0%	1353	37.3%
**T4**	82	2.0%	88	2.1%	79	2.2%	65	1.8%
**Unknown**	804	19.2%	835	19.9%	731	20.2%	739	20.4%
**Pathologic N Stage**								
**N0**	1853	44.2%	1852	44.1%	1406	38.8%	1441	39.7%
**N1**	1509	35.9%	1512	36.0%	1511	41.7%	1496	40.4%
**NX**	833	19.8%	833	19.8%	709	19.6%	699	19.3%
**Lymph nodes positive**								
**Negative**	1562	37.2%	1552	37.0%	1195	33.0%	1233	34.0%
**Positive**	1506	35.9%	1515	35.8%	1494	41.2%	1479	40.7%
**No LN examined**	1107	26.4%	1114	26.6%	911	25.1%	897	24.7%
**Unknown**	22	0.5%	22	0.5%	26	0.7%	17	0.5%
**Surgical approach**								
**Open**	1491	35.5%	1472	35.1%	1320	36.4%	1275	35.2%
**Minimally Invasive**	940	22.4%	945	22.5%	874	24.1%	891	24.6%
**No data before 2010**	1265	30.2%	1269	30.2%	719	19.7%	735	20.3%
**Unknown after 2010**	501	11.9%	511	12.2%	713	19.8%	725	20.4%

**Table 2:** Propensity-matched survival analysis by comparator and cancer type.

	N	5-yr Survival (%)	Median Survival (mo.)	*p* Value
Matched Intrahepatic (IHC)				
Group 1				0.319
Surgery Only	723	41.2	44.8	
XRT	723	38.8	38.9	
Group 2				0.445
Chemo	691	38.5	43.3	
XRT	691	37.7	38.7	
Matched Perihilar (PHC)				
Group 1				0.003
Surgery Only	693	24.7	26.3	
XRT	693	31.0	31.2	
Group 2				0.557
Chemo	445	27.0	26.5	
XRT	445	29.7	30.3	
Matched Distal (DCC)				
Group 1				0.015
Surgery Only	726	30.7	27.0	
XRT	726	33.4	33.7	
Group 2				0.298
Chemo	722	35.2	33.4	
XRT	722	31.8	32.9	
Matched Gallbladder (GBC)				
Group 1				0.05
Surgery Only	2055	33.8	26.6	
XRT	2055	32.5	30.2	
Group 2				0.001
Chemo	1768	31.5	24.6	
XRT	1768	32.6	30.2	

## Data Availability

Data are contained within the article.

## Supplementary Materials

The following supporting information can be downloaded at: https://www.mdpi.com/article/10.3390/cancers17030494/s1, Table S1: Propensity matched groups, intrahepatic cholangiocarcinoma (IHC); Table S2: Propensity matched groups, perihilar cholangiocarcinoma (PHC); Table S3: Propensity matched groups, distal cholangiocarcinoma (DCC); Table S4: Propensity matched groups, gallbladder cancer (GBC).
