# Supplementary material for: Defining the Role of Adjuvant Radiotherapy for Biliary Tract Cancers: A Site-Specific Propensity-Matched Analysis
Source: Cancers (Basel). 2025 Feb 2;17(3):494. doi: 10.3390/cancers17030494 (PMC11815919; doi:10.3390/cancers17030494)
Supplement: Supplementary file 1 [file cancers-17-00494-s001.zip › cancers-3428897-supplementary.pdf]

# Defining the Role of Adjuvant Radiotherapy for Biliary Tract Cancers: A Site-Specific Propensity-Matched Analysis

**Yongwoo David Seo [1,†], Belkacem Acidi [1,†], Andrew Newton [2], Antony Haddad [1], Yi-Ju Chiang [1], Rainna Coelho [3], Timothy E. Newhook [1], Ching-Wei D. Tzeng [1], Yun Shin Chun [1], Ethan B. Ludmir [4], Eugene J. Koay [4], Milind Javle [5], Jean Nicolas Vauthey [1] and Hop S. Tran Cao [1,*]**

[1] Department of Surgical Oncology, The University of Texas MD Anderson Cancer Center, Houston, TX 77030, USA
[2] Department of Surgical Oncology, Ochsner Health, New Orleans, LA 70112, USA
[3] Department of Surgery, HCA Houston Healthcare, University of Houston, Houston, TX 77204, USA
[4] Department of Radiation Oncology, The University of Texas MD Anderson Cancer Center, Houston, TX 77030, USA
[5] Department of Gastrointestinal Medical Oncology, The University of Texas MD Anderson Cancer Center, Houston, TX 77030, USA
† These authors contributed equally to this work.
* Correspondence: hstran@mdanderson.org; Tel.: +1-(713)-745-4670

Running Header: Outcomes of Adjuvant Chemoradiation in BTC

Keywords: Biliary tract cancer, adjuvant therapy, chemoradiotherapy, Liver cancer

*The authors do not have any relevant financial or commercial interests to disclose*

*The data used in this study are derived from a de-identified National Cancer Database file.*

## Supplementary

| Intrahepatic (IHC) | Matched group 1 (N=1446) | | | | | Matched group 2 (N=1382) | | | |
|---|---|---|---|---|---|---|---|---|---|
| | Surgery Only (n=723) | | XRT (n=723) | | | Chemo (n=691) | | XRT (n=691) | |
| | N | Col % | N | Col % | | N | Col % | N | Col % |
| **SEX** | | | | | | | | | |
| Male | 308 | 42,6 | 332 | 45,92 | | 306 | 44,28 | 314 | 45,44 |
| Female | 415 | 57,4 | 391 | 54,08 | | 385 | 55,72 | 377 | 54,56 |
| **Age** | | | | | | | | | |
| ≤ 65 | 397 | 54,91 | 378 | 52,28 | | 392 | 56,73 | 373 | 53,98 |
| > 65 | 326 | 45,09 | 345 | 47,72 | | 299 | 43,27 | 318 | 46,02 |
| **Race** | | | | | | | | | |
| White | 586 | 81,05 | 556 | 76,9 | | 540 | 78,15 | 533 | 77,13 |
| Black | 40 | 5,53 | 52 | 7,19 | | 35 | 5,07 | 52 | 7,53 |
| Hispanic | 37 | 5,12 | 64 | 8,85 | | 44 | 6,37 | 58 | 8,39 |
| Asian/Pacific Islander | 53 | 7,33 | 45 | 6,22 | | 65 | 9,41 | 43 | 6,22 |
| Others | 7 | 0,97 | 6 | 0,83 | | 7 | 1,01 | 5 | 0,72 |
| **Margin** | | | | | | | | | |
| R0 | 425 | 58,78 | 413 | 57,12 | | 407 | 58,9 | 404 | 58,47 |
| R1 | 158 | 21,85 | 159 | 21,99 | | 135 | 19,54 | 147 | 21,27 |
| RX | 140 | 19,36 | 151 | 20,89 | | 149 | 21,56 | 140 | 20,26 |
| **Pathologic T stage** | | | | | | | | | |
| T1 | 166 | 22,96 | 174 | 24,07 | | 128 | 18,52 | 139 | 20,12 |
| T2 | 165 | 22,82 | 171 | 23,65 | | 175 | 25,33 | 174 | 25,18 |
| T3 | 106 | 14,66 | 95 | 13,14 | | 101 | 14,62 | 94 | 13,6 |
| T4 | 10 | 1,38 | 11 | 1,52 | | 7 | 1,01 | 10 | 1,45 |
| Unknown | 276 | 38,17 | 272 | 37,62 | | 280 | 40,52 | 274 | 39,65 |
| **Pathologic N stage** | | | | | | | | | |
| N0 | 377 | 52,14 | 372 | 51,45 | | 347 | 50,22 | 348 | 50,36 |
| N1 | 96 | 13,28 | 98 | 13,55 | | 113 | 16,35 | 108 | 15,63 |
| NX | 250 | 34,58 | 253 | 34,99 | | 231 | 33,43 | 235 | 34,01 |
| **Lymph nodes postitve** | | | | | | | | | |
| Negative | 312 | 43,15 | 317 | 43,85 | | 290 | 41,97 | 296 | 42,84 |
| Positive | 97 | 13,42 | 96 | 13,28 | | 114 | 16,5 | 106 | 15,34 |
| No LN examined | 312 | 43,15 | 307 | 42,46 | | 283 | 40,96 | 286 | 41,39 |
| Unknown | 2 | 0,28 | 3 | 0,41 | | 4 | 0,58 | 3 | 0,43 |
| **Surgical approach** | | | | | | | | | |
| Open | 293 | 40,53 | 304 | 42,05 | | 287 | 41,53 | 287 | 41,53 |
| Minimally Invasive | 78 | 10,78 | 83 | 11,48 | | 70 | 10,13 | 83 | 12,01 |
| No data before 2010 | 227 | 31,4 | 214 | 29,6 | | 181 | 26,19 | 173 | 25,04 |
| Unknown after 2010 | 125 | 17,29 | 122 | 16,87 | | 153 | 22,14 | 148 | 21,42 |

**Supplementary Table S1: Propensity matched groups, intrahepatic cholangiocarcinoma (IHC)**

| Perihilar (PHC) | Matched group 1 (N=1386) | | | | | Matched group 2 (N=890) | | | |
|---|---|---|---|---|---|---|---|---|---|
| | Surgery Only (n=693) | | XRT (n=693) | | | Chemo (n=445) | | XRT (n=445) | |
| | N | Col % | N | Col % | | N | Col % | N | Col % |
| **SEX** | | | | | | | | | |
| Male | 424 | 61,18 | 435 | 62,77 | | 278 | 62,47 | 276 | 62,02 |
| Female | 269 | 38,82 | 258 | 37,23 | | 167 | 37,53 | 169 | 37,98 |
| **Age** | | | | | | | | | |
| ≤ 65 | 308 | 44,44 | 317 | 45,74 | | 219 | 49,21 | 231 | 51,91 |
| > 65 | 385 | 55,56 | 376 | 54,26 | | 226 | 50,79 | 214 | 48,09 |
| **Race** | | | | | | | | | |
| White | 532 | 76,77 | 535 | 77,2 | | 334 | 75,06 | 329 | 73,93 |
| Black | 49 | 7,07 | 61 | 8,8 | | 33 | 7,42 | 37 | 8,31 |
| Hispanic | 48 | 6,93 | 41 | 5,92 | | 40 | 8,99 | 41 | 9,21 |
| Asian/Pacific Islander | 56 | 8,08 | 44 | 6,35 | | 27 | 6,07 | 28 | 6,29 |
| Others | 8 | 1,15 | 12 | 1,73 | | 11 | 2,47 | 10 | 2,25 |
| **Margin** | | | | | | | | | |
| R0 | 437 | 63,06 | 445 | 64,21 | | 298 | 66,97 | 298 | 66,97 |
| R1 | 135 | 19,48 | 134 | 19,34 | | 78 | 17,53 | 80 | 17,98 |
| RX | 121 | 17,46 | 114 | 16,45 | | 69 | 15,51 | 67 | 15,06 |
| **Pathologic T stage** | | | | | | | | | |
| T1 | 41 | 5,92 | 53 | 7,65 | | 29 | 6,52 | 29 | 6,52 |
| T2 | 301 | 43,43 | 281 | 40,55 | | 178 | 40 | 193 | 43,37 |
| T3 | 232 | 33,48 | 220 | 31,75 | | 165 | 37,08 | 163 | 36,63 |
| T4 | 31 | 4,47 | 34 | 4,91 | | 25 | 5,62 | 15 | 3,37 |
| Unknown | 88 | 12,7 | 105 | 15,15 | | 48 | 10,79 | 45 | 10,11 |
| **Pathologic N stage** | | | | | | | | | |
| N0 | 349 | 50,36 | 357 | 51,52 | | 191 | 42,92 | 197 | 44,27 |
| N1 | 287 | 41,41 | 285 | 41,13 | | 214 | 48,09 | 222 | 49,89 |
| NX | 57 | 8,23 | 51 | 7,36 | | 40 | 8,99 | 26 | 5,84 |
| **Lymph nodes postitve** | | | | | | | | | |
| Negative | 338 | 48,77 | 338 | 48,77 | | 182 | 40,9 | 190 | 42,7 |
| Positive | 287 | 41,41 | 285 | 41,13 | | 214 | 48,09 | 222 | 49,89 |
| No LN examined | 63 | 9,09 | 65 | 9,38 | | 43 | 9,66 | 31 | 6,97 |
| Unknown | 5 | 0,72 | 5 | 0,72 | | 6 | 1,35 | 2 | 0,45 |
| **Surgical approach** | | | | | | | | | |
| Open | 243 | 35,06 | 234 | 33,77 | | 177 | 39,78 | 162 | 36,4 |
| Minimally Invasive | 53 | 7,65 | 51 | 7,36 | | 35 | 7,87 | 40 | 8,99 |
| No data before 2010 | 361 | 52,09 | 368 | 53,1 | | 173 | 38,88 | 176 | 39,55 |
| Unknown after 2010 | 36 | 5,19 | 40 | 5,77 | | 60 | 13,48 | 67 | 15,06 |

**Supplementary Table S2: Propensity matched groups, perihilar cholangiocarcinoma (PHC)**

| Distal (DCC) | Matched group 1 (N=1452) | | | | Matched group 2 (N=1444) | | | |
|---|---|---|---|---|---|---|---|---|
| | Surgery Only (n=726) | | XRT (n=726) | | Chemo (722) | | XRT (n=722) | |
| | N | Col % | N | Col % | N | Col % | N | Col % |
| **SEX** | | | | | | | | |
| **Male** | 452 | 62,26 | 462 | 63,64 | 451 | 62,47 | 448 | 62,05 |
| **Female** | 274 | 37,74 | 264 | 36,36 | 271 | 37,53 | 274 | 37,95 |
| **Age** | | | | | | | | |
| **≤ 65** | 299 | 41,18 | 299 | 41,18 | 352 | 48,75 | 337 | 46,68 |
| **> 65** | 427 | 58,82 | 427 | 58,82 | 370 | 51,25 | 385 | 53,32 |
| **Race** | | | | | | | | |
| **White** | 562 | 77,41 | 564 | 77,69 | 548 | 75,9 | 565 | 78,25 |
| **Black** | 56 | 7,71 | 61 | 8,4 | 67 | 9,28 | 61 | 8,45 |
| **Hispanic** | 57 | 7,85 | 56 | 7,71 | 53 | 7,34 | 55 | 7,62 |
| **Asian/Pacific Islander** | 44 | 6,06 | 37 | 5,1 | 43 | 5,96 | 34 | 4,71 |
| **Others** | 7 | 0,96 | 8 | 1,1 | 11 | 1,52 | 7 | 0,97 |
| **Margin** | | | | | | | | |
| **R0** | 542 | 74,66 | 527 | 72,59 | 563 | 77,98 | 562 | 77,84 |
| **R1** | 95 | 13,09 | 111 | 15,29 | 83 | 11,5 | 84 | 11,63 |
| **RX** | 89 | 12,26 | 88 | 12,12 | 76 | 10,53 | 76 | 10,53 |
| **Pathologic T stage** | | | | | | | | |
| **T1** | 34 | 4,68 | 44 | 6,06 | 26 | 3,6 | 30 | 4,16 |
| **T2** | 160 | 22,04 | 157 | 21,63 | 133 | 18,42 | 138 | 19,11 |
| **T3** | 454 | 62,53 | 443 | 61,02 | 503 | 69,67 | 499 | 69,11 |
| **T4** | 23 | 3,17 | 25 | 3,44 | 24 | 3,32 | 19 | 2,63 |
| **Unknown** | 55 | 7,58 | 57 | 7,85 | 36 | 4,99 | 36 | 4,99 |
| **Pathologic N stage** | | | | | | | | |
| **N0** | 328 | 45,18 | 319 | 43,94 | 256 | 35,46 | 256 | 35,46 |
| **N1** | 379 | 52,2 | 386 | 53,17 | 450 | 62,33 | 453 | 62,74 |
| **NX** | 19 | 2,62 | 21 | 2,89 | 16 | 2,22 | 13 | 1,8 |
| **Lymph nodes postitve** | | | | | | | | |
| **Negative** | 322 | 44,35 | 312 | 42,98 | 250 | 34,63 | 250 | 34,63 |
| **Positive** | 379 | 52,2 | 387 | 53,31 | 446 | 61,77 | 452 | 62,6 |
| **No LN examined** | 24 | 3,31 | 27 | 3,72 | 20 | 2,77 | 17 | 2,35 |
| **Unknown** | 1 | 0,14 | . | . | 6 | 0,83 | 3 | 0,42 |
| **Surgical approach** | | | | | | | | |
| **Open** | 487 | 67,08 | 491 | 67,63 | 454 | 62,88 | 429 | 59,42 |
| **Minimally Invasive** | 104 | 14,32 | 106 | 14,6 | 102 | 14,12 | 113 | 15,65 |
| **No data before 2010** | 56 | 7,71 | 60 | 8,26 | 23 | 3,19 | 26 | 3,6 |
| **Unknown after 2010** | 79 | 10,88 | 69 | 9,5 | 143 | 19,81 | 154 | 21,33 |

**Supplementary Table S3: Propensity matched groups, distal cholangiocarcinoma (DCC)**

| Gallbladder (GBC) | Matched group 1 (N=4110) | | | | Matched group 2 (N=3536) | | | |
|---|---|---|---|---|---|---|---|---|
| | Surgery Only (n=2055) | | XRT (n=2055) | | Chemo (n=1768) | | XRT (n=1768) | |
| | N | Col % | N | Col % | N | Col % | N | Col % |
| **SEX** | | | | | | | | |
| Male | 640 | 31,14 | 637 | 31 | 527 | 29,81 | 540 | 30,54 |
| Female | 1415 | 68,86 | 1418 | 69 | 1241 | 70,19 | 1228 | 69,46 |
| **Age** | | | | | | | | |
| ≤ 65 | 809 | 39,37 | 817 | 39,76 | 780 | 44,12 | 784 | 44,34 |
| > 65 | 1246 | 60,63 | 1238 | 60,24 | 988 | 55,88 | 984 | 55,66 |
| **Race** | | | | | | | | |
| White | 1387 | 67,49 | 1354 | 65,89 | 1194 | 67,53 | 1135 | 64,2 |
| Black | 276 | 13,43 | 306 | 14,89 | 254 | 14,37 | 282 | 15,95 |
| Hispanic | 240 | 11,68 | 252 | 12,26 | 208 | 11,76 | 216 | 12,22 |
| Asian/Pacific Islander | 122 | 5,94 | 114 | 5,55 | 89 | 5,03 | 109 | 6,17 |
| Others | 30 | 1,46 | 29 | 1,41 | 23 | 1,3 | 26 | 1,47 |
| **Margin** | | | | | | | | |
| R0 | 1378 | 67,06 | 1403 | 68,27 | 1166 | 65,95 | 1166 | 65,95 |
| R1 | 296 | 14,4 | 287 | 13,97 | 252 | 14,25 | 252 | 14,25 |
| RX | 381 | 18,54 | 365 | 17,76 | 350 | 19,8 | 350 | 19,8 |
| **Pathologic T stage** | | | | | | | | |
| T1 | 40 | 1,95 | 50 | 2,43 | 29 | 1,64 | 35 | 1,98 |
| T2 | 968 | 47,1 | 939 | 45,69 | 739 | 41,8 | 738 | 41,74 |
| T3 | 644 | 31,34 | 647 | 31,48 | 610 | 34,5 | 590 | 33,37 |
| T4 | 18 | 0,88 | 18 | 0,88 | 23 | 1,3 | 21 | 1,19 |
| Unknown | 385 | 18,73 | 401 | 19,51 | 367 | 20,76 | 384 | 21,72 |
| **Pathologic N stage** | | | | | | | | |
| N0 | 799 | 38,88 | 804 | 39,12 | 612 | 34,62 | 640 | 36,2 |
| N1 | 747 | 36,35 | 743 | 36,16 | 734 | 41,52 | 703 | 39,76 |
| NX | 509 | 24,77 | 508 | 24,72 | 422 | 23,87 | 425 | 24,04 |
| **Lymph nodes postitve** | | | | | | | | |
| Negative | 588 | 28,61 | 585 | 28,47 | 473 | 26,75 | 497 | 28,11 |
| Positive | 743 | 36,16 | 734 | 35,72 | 720 | 40,72 | 699 | 39,54 |
| No LN examined | 708 | 34,45 | 722 | 35,13 | 565 | 31,96 | 563 | 31,84 |
| Unknown | 16 | 0,78 | 14 | 0,68 | 10 | 0,57 | 9 | 0,51 |
| **Surgical approach** | | | | | | | | |
| Open | 468 | 22,77 | 443 | 21,56 | 402 | 22,74 | 397 | 22,45 |
| Minimally Invasive | 705 | 34,31 | 705 | 34,31 | 667 | 37,73 | 655 | 37,05 |
| No data before 2010 | 621 | 30,22 | 627 | 30,51 | 336 | 19 | 350 | 19,8 |
| Unknown after 2010 | 261 | 12,7 | 280 | 13,63 | 363 | 20,53 | 366 | 20,7 |

**Supplementary Table S4: Propensity matched groups, gallbladder cancer (GBC)**
